# The Role of Polyisobutylene-Bis-Succinimide (PIBSI) Dispersants in Lubricant Oils on the Deposit Control Mechanism

**DOI:** 10.3390/polym17081041

**Published:** 2025-04-11

**Authors:** Erhan Özdemir, Esra Kan, Binbin Guo, Eugene Pashkovski, Anil Agiral, Erol Yildirim

**Affiliations:** 1 Department of Chemistry, Middle East Technical University, 06800 Ankara, Turkey; 2 Department of Polymer Science and Technology, Middle East Technical University, 06800 Ankara, Turkey; 3 The Lubrizol Corporation, Wickliffe, OH 44092, USA; 4 Department of Micro and Nanotechnology, Middle East Technical University, 06800 Ankara, Turkey

**Keywords:** dispersant, multiscale modeling, molecular dynamics simulation, coarse-grained simulations

## Abstract

Molecular modeling calculations for the design and improvement of next-generation additives for motor oils have reached a level that can support and improve experimental results. The regulation of insoluble sludge nanoparticle aggregations within oil and on engine pistons is a critical performance metric for lubricant oil additives. There is a general agreement regarding the mechanism of deposit formation which is attributed to the self-aggregation of nano-sized carbon rich insoluble structures. Dispersants are a primary category of additives employed to inhibit aggregation in lubricant formulations. Along with the base oil, they are crucial in dispersing and stabilizing insoluble particles to manage the formation of deposits. In this study, multiscale modeling methods were used to elucidate molecular mechanism of deposit control via polyisobutylene-bis-succinimide (PIBSI) dispersants by using density functional theory (DFT), molecular dynamics (MD) simulations of cells constructed by statistical sampling of molecular configurations, and coarse-grained (CG) simulations. The aim of this study was to understand the role of different groups such as succinimide, amine center, and two polyisobutylene (PIB) tails in PIBSI dispersants. It was demonstrated that the mechanism of deposit control by the polymer-based PIBSI dispersant can be elucidated through the interactions among various constituents, including hydrogen bonding and hydrophilic–hydrophobic interactions. We showed that sludge type nanoparticle aggregation is mitigated by intercalation of polar amine central groups of dispersant between the nanoparticles followed by the extension of two hydrophobic PIB chains into the oil phase that decreases coalesce further by forming a hydrophobic repulsive layer.

## 1. Introduction

From ancient times, lubricants have evolved into a substantial global industry [1,2]. The progression of contemporary engine and transmission technologies would have been unattainable without the advancements in lubricant additive chemistry and lubricant formulations. Regardless of their application, the oils employed for the lubrication of internal combustion engines are exposed to a vast and diverse array of temperatures and operating conditions [3,4]. The insoluble deposits are formed as a by-product of incomplete fuel oxidation. Deposits are formed due to insufficient and unfinished combustion and they add up constituting aggregates, which can clog the filters and certain parts of the engine [5,6,7]. These insoluble deposits, namely soot and sludge, are carbon-rich particles formed as a by-product of incomplete fuel oxidation [5,6,8].

Both detergents and dispersants have a similar role in dispersing insoluble particles in the engine oils [9,10,11,12]. Dispersants as lubricant additives function to distribute and maintain the suspension of soot and sludge particles [5]. This helps prevent the creation of deposits at different temperatures in engine cylinders and rings [7,13,14]. The primary roles of these additivies such as dispersants are maintaining engine cleanliness, neutralizing acids, solubilizing and dispersing sludge particles in oil, and mitigating deposit formation [14,15,16]. While the precise working mechanism is not fully comprehended, it is accepted that dispersant- and detergent-type oil additives can function in two distinct manners: they can either suspend particles and precursors of deposits in the oil to mitigate deposit formation or they can form a protective layer on the metal surface to obstruct deposit formation [5,14,17,18].

Examples of widely acknowledged detergents and dispersants encompass sulfonates (including metal, ashless, or overbased varieties), salicylates, alkyl phenolates, carboxylates, polyisobutylene succinimides, Mannich adducts, polyethylene glycol esters, polyol poly-(12-hydroxy stearic acid), phosphates, and phosphonates, each characterized by their distinct structural compositions [5,9,11,13,19,20].

**Figure 1:**
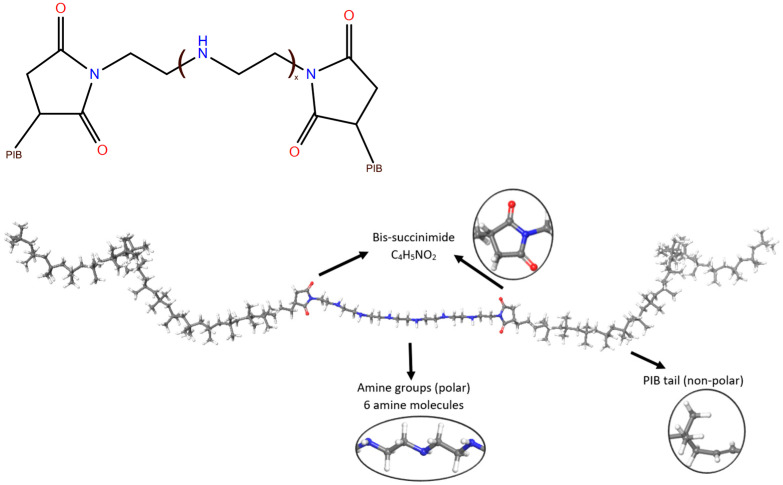
PIBSI dispersant molecular structure and model.

Bis-succinimide and tris-succinimide, with polyisobutylene (PIB) tails at 1000–2000 g/mol MW, are the most common type of polymeric dispersants [21]. The type of amine group in center, PIB chain size, and amount of succinimides (bis vs. tris) can all be controlled for performance. The ratio of a typical polyamine to PIBSA changes from 1:1 to 2:1 to 3:1, resulting in mono-, bis, and tris-succinimides, respectively. The primary amine preferentially reacts with the PIBSI unit and water in each case to create an imide group. After all the primary nitrogen atoms are consumed in the tris model, a secondary amine can open up the additional anhydride group to generate an amide. In fact, depending on the polyamine utilized, branching and cyclic species are present in varying proportions [20,22,23,24]. The model used in this study for PIBSI is given in Figure 1.

The working mechanisms of polymeric dispersant were speculated at molecular level, and aggregation has never been studied theoretically in the literature. This study aims to elucidate the deposit control mechanisms of polymeric PIBSI type dispersants. Multiscale modeling methods are used to elucidate the mechanism of deposit formation in engine oil. In addition, control and mitigation of deposit formation via dispersants is also simulated. Developing a reliable model of the engine environment will enable future optimization of dispersant structures for enhanced performance in deposit control.

This study is started by the construction of an insoluble sludge type deposit model. The exact molecular structure of the insoluble sludge-type deposit is not known. While there are multiscale modeling studies on oils and dyes, this study is one of the first few in which an insoluble sludge-type deposit model is constructed and aggregation of these insoluble deposit models is demonstrated [25,26,27,28,29]. In addition, within the scope of this study, models for base oil and dispersant are developed for all-atom and CG MD simulations. The development of aggregates of insoluble deposits and the prevention of aggregation by model dispersants are studied in this way. We investigated the formation of insoluble deposit aggregates and their prevention using the PIBSI dispersant model.

## 2. Materials and Methods

Experimental results served as the guiding framework for the computational experiments. All experimental results on lubricant oil recipe and insoluble nanoparticles were provided by Lubrizol Corporation. Experimental data provided by Lubrizol, such as the C/O ratio in insoluble nanoparticles, were used for the computational studies. The main experimental results were nanoparticle agglomeration in both drain oil and on piston grooves with similar molecular structure, which forms larger-sized deposits in the former. It was observed that these deposits accumulate in the piston grooves, clog them over time, and prevent engine running. Experimental results showed that detergents and dispersants can mitigate this problem. In the case of lubricant, oil samples from the engine environment were examined, characterized by FTIR, XRD, XPS, DLS, Zeta Potential, Electron microscopy, Focused Beam Reflectance, Probe Microscopy, and elemental analysis by Lubrizol [25].

**Figure 2:**
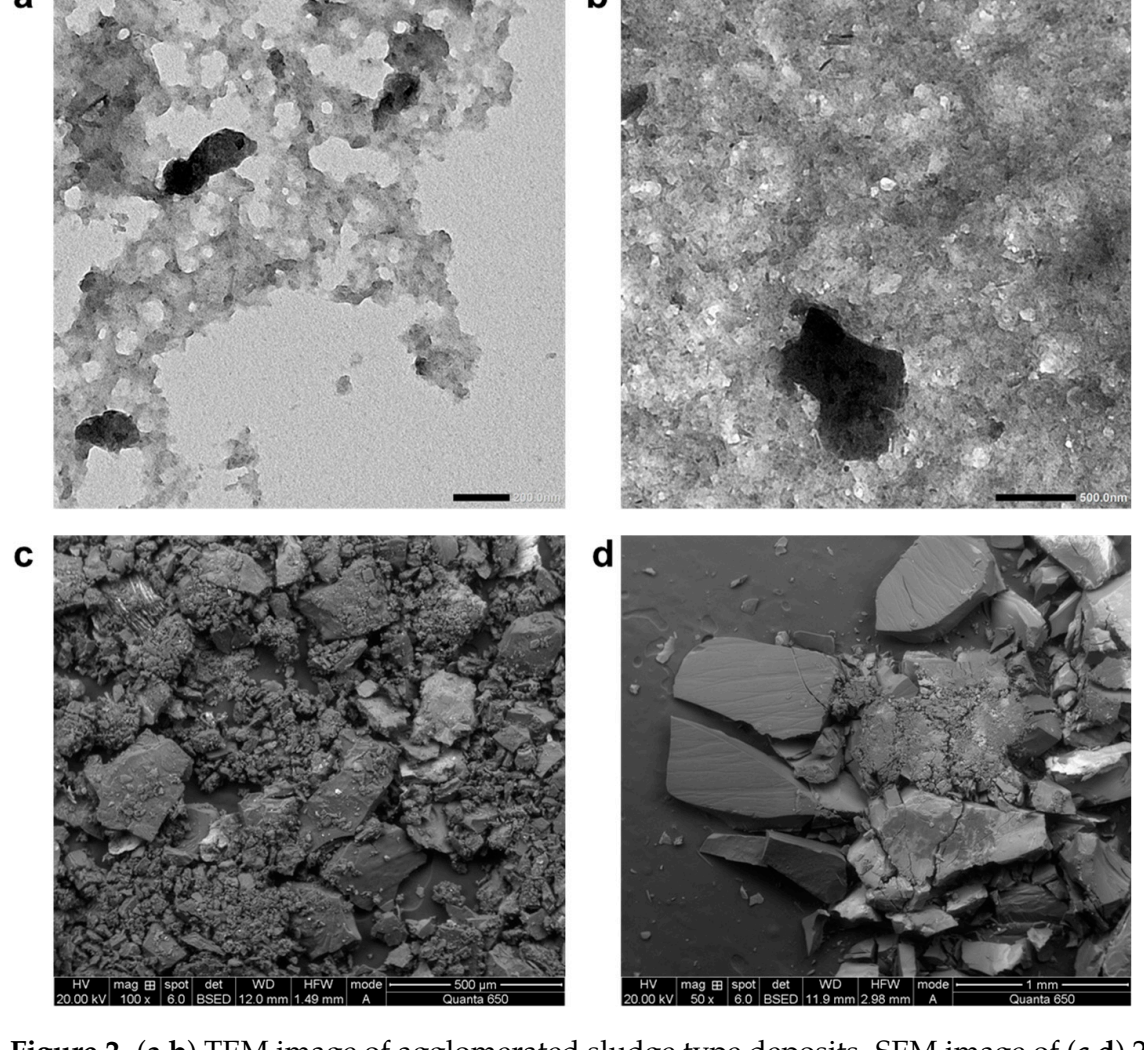
(**a**,**b**) TEM image of agglomerated sludge type deposits. SEM image of (**c**,**d**) 2nd and 3rd land deposits and c insoluble deposits from drain oil.

In Figure 2, TEM and SEM images for 2nd and 3rd land deposit and drain oil insolubles are given. Similar morphological structures were observed from the deposit formation and drain insoluble particle images. XRD phase data indicate that deposits are primarily amorphous carbon with traces of silicon carbide. Using elemental analysis and XPS, it was observed that the insoluble nanoparticle surface is mostly composed of carbon and oxidized carbons in amorphous structure. Elemental composition analysis land deposits have 72% carbon 25% oxygen as mol ratio. The remaining portion is mostly composed of magnesium, phosphorus, sulfur, and very small proportions of zinc, calcium, sodium, aluminum, copper, and silicon. For the drain insoluble particles, elemental composition pointed to the fact that there is 74% carbon and 23% oxygen judging by number of atoms which was used in the modeling step of this study. There is a small proportion of other elements [25]. All the characterization methods pointed out that samples from the engine environment and drain insoluble particles, which were causing deposits on the engine pistons, have similar molecular structures. It is proposed that aggregation of drain insoluble nanoparticles creates larger insolubles in the engine oil, which form deposits that clog the piston lands. Moving forward, controlling the generation and colloidal stabilization of insolubles at initial stages can be the key for deposit control.

For further understanding and elucidating the role of dispersant, the difference was shown previously using TEM [5]. When extra dispersant was added to the oil, dispersion of insolubles showed significant improvement when adding extra dispersant. In the base oil, the addition of extra dispersant resulted in reduction in the nanoparticle aggregation [24,30,31,32].

### 2.1. Molecular Modeling Methods

#### 2.1.1. Density Functional Theory Calculations

All potential interaction energies between components were computed using B3LYP/6-31+g(d) level DFT calculations. Dispersants were modeled by amine, bis-succinimide (bis-suc), and PIB groups. The surfaces of insoluble nanoparticles were represented by carboxylic acid-substituted (Nacid), ketone-substituted (Nketone), alcohol (Nalcohol), and dialcohol (Nalcohol2) substituted branched alkane groups. The base oil was represented by alkyl groups, while the contributions from insignificant branching and alkene groups were disregarded in DFT calculations.

#### 2.1.2. All-Atom MD Simulations

Prior to the all-atom MD simulations, initial all-atom structures for the insoluble sludge-type deposit (which is called “nanoparticles”), Group II base oil with 24 carbon atoms, including one alkene group and two short branches (Figure S1), and bis-succinimide dispersant models were constructed.

#### 2.1.3. Insoluble Sludge Nanoparticle Model

**Figure 3:**
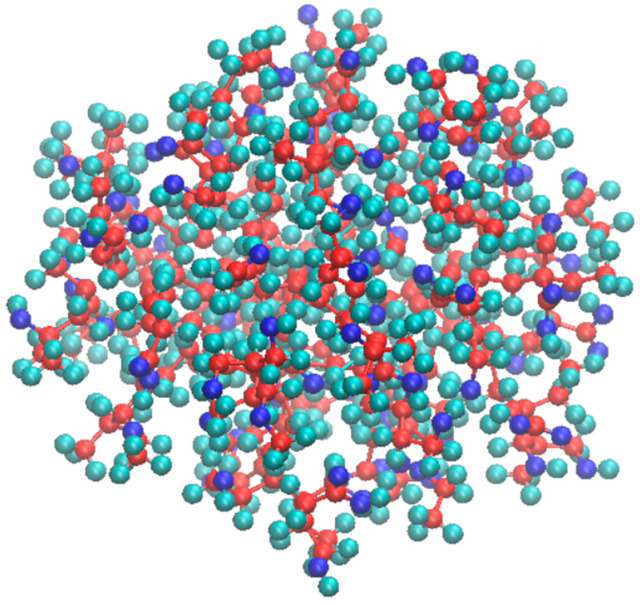
Insoluble deposit model. Molecular formula: C_300_H_599_O_100_. 75% C, 25% O based on the experimental results provided by the Lubrizol company. Carbon atoms are red; oxygen atoms are blue and hydrogen atoms are green in color.

A significant challenge in modeling the study was modeling the ultrafine insoluble nanoparticles that aggregate to form engine deposits. Due to the lack of molecular-level information about the structure of these aggregated particles in the literature, the model was manually constructed based on experience and experimental data. From the experimental results provided by Lubrizol Corporation, it is known that this nanoparticle may have many functional groups such as primary–secondary–tertiary alcohols, ketone, aldehyde, carboxylic acid, ester, and ether groups. The insoluble deposit was modeled using C and O ratios based on the experimental elemental composition analysis results. For the drain insoluble, the mole ratios of C and O were found to be 74% and 23%, respectively. Keeping these values in modeling, the insoluble deposit model was constructed using 75% C and 25% O, where minor elements Mg, P, S and others in smaller portions were ignored. A spherical-shaped nanoparticle with a diameter of about 2.2 nm was modeled as the smallest unit of the insoluble deposit as shown in Figure 3 [25]. The chemical formula and the molecular weight of this insoluble deposit model were C_300_H_599_O_100_ and 5766.57 g/mol, respectively.

In order to mimic the aggregation process in the engine pistons, polar functional groups were positioned on the surface. The functional groups were connected to each other from the center of the sphere to the surface via chemical bonds. It was manually modeled to ensure it meets several criteria: avoiding errors caused by close bond distances, satisfying convergence requirements for both force and energy, maintaining negative total free energy, being compatible with the chosen force field in computational methods, aligning with the experimental C/O ratio, and ensuring internal consistency within the structure. To verify the validity of the initial structure, energy reduction was achieved through annealing (heating–cooling) cycles, which resulted in a stable amorphous nanoparticle. In the modeled nanoparticle, oxygen was primarily concentrated on the surface and was designed in accordance with the elemental analysis, as illustrated in Figure 3 [25]. Construction of the base oil, dispersant molecules, and simulation cells are provided in Supporting Information.

#### 2.1.4. Validation of Computational Method and Force Field Approach

Initially, base oil molecules were incorporated into the cell to verify the accuracy of the base oil structure and methodology. The experimental density of the base oil, reported by Lubrizol Corporation is between 0.86 and 0.87 g/cm^3^, and this value was used as the validation parameter. A total of 50,000 steps of geometry and cell parameter optimizations were conducted for the cell, beginning with an initial density of 0.5 g/cm^3^ and employing stringent convergence criteria for force and displacement. Following the geometry optimization, the density of the base oil within the unit cell increased from 0.50 g/cm^3^ to 0.85 g/cm^3^ and subsequently to 0.86 g/cm^3^ after the heating–cooling cycles. Therefore, the validity of the base oil structure and computational methodology was confirmed, as the experimental density provided by the company aligns with the all-atom molecular dynamics simulations. After preparation and validation of system components, solubility properties and polarity descriptors were calculated by QSAR methods.

#### 2.1.5. All-Atom Molecular Dynamics Simulation Procedure

Simulations were carried out using LAMMPS (3 March 2020 version). Polymer consistent force field (PCFF) was used for bonded and non-bonded interactions where 9−6 Lennard–Jones (LJ) was used for short-ranged repulsion–dispersion interactions. Particle–particle particle–mesh solver (PPPM) was used for long-range electrostatic interactions. Data visualization and calculations were carried out with VMD visualizer. The simulation box has periodic boundary conditions (PBCs) in the x, y, and z directions. Initial structures were minimized using the steepest descent algorithm integrated into the LAMMPS package. The stopping tolerance for energy is 1.0 × 10^−4^ (arbitrary units) and the stopping tolerance for force is 1.0 × 10^−6^ (kcal/mol)/Å. As a general procedure in the minimization of the initial structures, iterations are terminated when one of these tolerances is satisfied for the energy or force in subsequent time steps. The maximum number of iterations was set as 1000 and the maximum number of force and energy evaluations was set as 10.000, at which minimization is terminated. The time step was 1 fs. An NVT ensemble with Nose–Hoover thermostat at 423 K were used which is the average engine oil temperature. Temperature was kept constant at 423 K for a 6.0 ns total simulation time.

### 2.2. Coarse-Grained MD Simulations

Similar to the all-atom MD simulations, CG models of insoluble deposit, base oil, and dispersant for the Martini 2.0 force field were constructed before CGMD simulations as described in detail in Supplementary Materials.

#### 2.2.1. Construction of the Coarse-Grained Models

Scienomics Atomeso Converter was utilized for mapping atoms of the deposit model, base oil, and dispersant into CG beads for CGMD simulations with a Martini 2.0 force field. Once the bead coordinates were gathered from the Scienomics Atomeso Converter, Python 3.9.18 scripts and Packmol v20.10.0 and Moltemplate 2.20.10 software were used for the preparation of LAMMPS input files. Data visualization and calculations were carried out with VMD. More details on the construction of insoluble deposit models with 2.2, 3.2, and 4.4 nm diameters, base oil, and dispersant CG models and simulation cells are provided in Supplementary Materials.

#### 2.2.2. Coarse-Grained Simulation Procedure

Steric clashes were eliminated using the steepest-descent minimization algorithm integrated within the LAMMPS package. Cell optimization was conducted for 4.0 ns in the NPT ensemble at 300 K, followed by a 16.0 ns simulation in the NVT ensemble at 423 K, employing a time step of 10 fs. Bead positions were documented in a trajectory file at 20 ps intervals. Coordinates of the last 4.0 ns were used for RDF analysis for which particle positions were relatively stable.

## 3. Results and Discussion

### 3.1. Density Functional Theory Calculation Results

**Figure 4:**
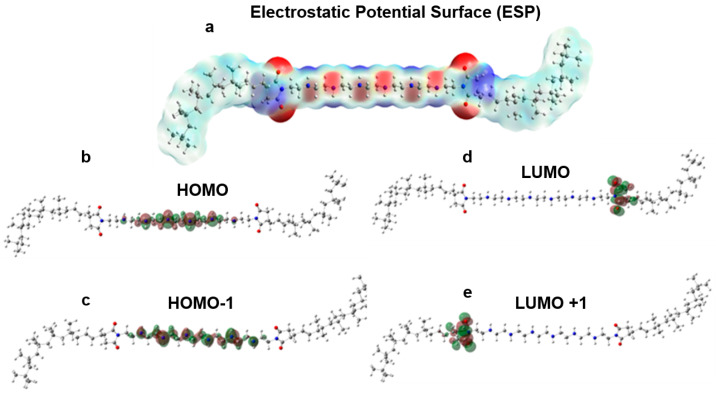
(**a**) Electrostatic Surface Potential, (**b**) HOMO, (**c**) HOMO-1, (**d**) LUMO, and (**e**) LUMO+1 structures of dispersant molecule.

PIBSI dispersants have three main groups: two PIB tails, the bis-succinimide group (C_4_H_5_NO_2_) bridges, and an amine central group. Electrostatic Potential Surface (ESP) and frontier orbitals are shown in Figure 4a. On the ESP surface, electron-rich to electron-poor regions were depicted from red to blue, respectively. As indicated in Figure 4a, especially oxygen atoms in succinimide and nitrogen atoms in amines have the highest electron density depicted in red. In addition, molecular structure, electrostatic potential surface, HOMO, and LUMO orbitals of polyisobutylene–bis-succinimide dispersant demonstrate their role even at molecular level. Amine hydrogens and carbons in bis-succinimide are the electron-deficient groups. According to these results, succinimide and amine groups are polar structures; however, poly-isobutylene tails are highly non-polar tails. Hydrophobicity and non-polar character are also valid for the base oil structures. HOMO–LUMO orbitals are also concentrated on the amine groups and bis-succinimide, respectively, which is a strong indication for potential polar interactions as well as susceptibility towards nucleophilic–electrophilic attacks via those groups.

Amine center was built with six amine molecules connected to each other via ethyl groups as given in Figure 1. Partial atomic charges are calculated according to DFT calculations at the B3LYP/6-311 level that supports polarity of the central amine–bis-succinimide group.

**Figure 5:**
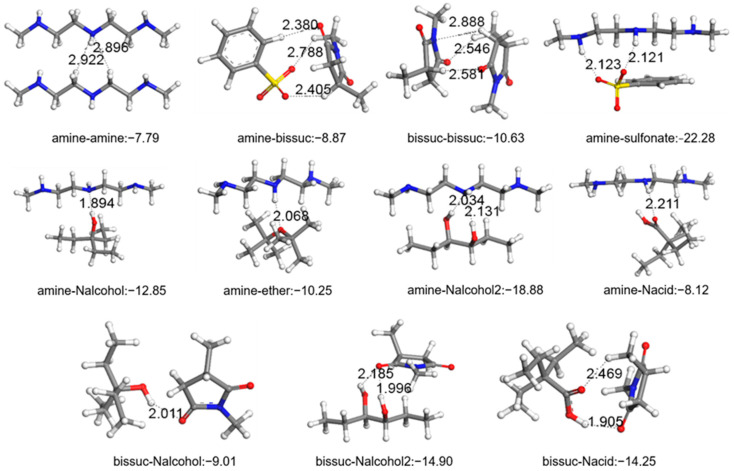
DFT calculation results for lowest energy structures and interaction energies of dispersant components with other constituents. Carbons are grey; oxygens are red, nitrogen are blue and hydrogen atoms white in color.

Pairwise interaction energies calculated by DFT calculations between alkyl tail, polar polyisobutylene groups, polar amine center of dispersant and all possible functional groups were found on the surface of an insoluble deposit as demonstrated in Figure 5. Calculated interaction energies are listed in Table 1. The highest interaction energy was calculated between the central amine group of the dispersant and alcohol functional groups which can exist on the surface of an insoluble deposit. These results showed that the strongest interactions were the ones formed between polar functional groups of the dispersant with the polar functional groups on the insoluble nanoparticles.

Alkyl groups showed the weakest interactions with the functional groups on the nanoparticle surface. Alkyl groups showed higher interactions with alkyl and PIB compared to their interactions with these functional groups. Although alkyl groups had relatively high interactions with amine groups, the interaction of these amine groups with functional groups on the nanoparticle surface was much higher. It should be noted that a number of interactions were not considered here; hence the alkyl–alkyl interactions are dominant due to the higher ratio of the base oil in the system.

It can also be seen Table 1 that there are strong-interaction amine groups of the dispersant and sulfonate head group of the sulfonate-based detergent, which is another possible additive in lubricant oils. This result is a novel output, indicating that deposit control performance of the dispersant and detergent additives can affect each other due to antagonistic or synergistic effects. In summary, our calculations showed that the strongest interactions are the ones formed by bis-succinimide groups and amine groups on dispersants with the functional groups on insoluble nanoparticles.

### 3.2. Solubility Parameter Calculations

Following the modeling of all structures within the oil, the initial step involves calculating the interactions and mixing of these components. The most straightforward and effective approach to accomplish this is by determining the solubility parameters. Solubility parameters that are similar in value tend to promote mixing, whereas those that are dissimilar generally do not mix. The calculation of solubility parameters for all structures was carried out in three stages. In the first stage, 20 amorphous periodic cells were constructed, with 30 to 60 molecules packed into each cell depending on cell size. In the second stage, geometry optimizations for all the cells were conducted for a minimum of 5000 steps until the energy and force convergence criteria were met. In the third stage, the average solubility parameters (including van der Waals and electrostatic contributions) were calculated for all cells Table 2.

As indicated previously, the polar groups of the dispersant are amine and succinimide and the non-polar groups are the polyisobutylene tails. During calculation of the solubility parameter, vdW contribution and electrostatic contribution to the solubility were also calculated separately. Electrostatic interactions were important for polar groups of the molecules. The non-polar part of the dispersant had almost zero electrostatic contribution. Next, the base oil had also almost zero electrostatic contribution due to its non-polar structure. It was determined that the solubility parameters of the non-polar group of the dispersant and oil as well as the polar group of the dispersant and sulfonate head group had values close to each other. These results promoted the idea that the non-polar tail of the dispersant may extend into the base oil, while amine and succinimide prefer to interact with the polar surface of the nanoparticle. The solubility parameter of the nanoparticle was not calculated due to the inability to construct a sufficient number of nanoparticle samples within multiple cells. However, it is recognized that the surface of the nanoparticle comprises oxygen-rich functional polar groups.

The hydrophobicity and surface properties of the components were assessed using various parameters, including the octanol–water partition coefficient (AlogP), solvent-accessible surface area (SASA), total polar surface area (TPSA), total apolar surface area (TASA), relative polar surface area (RPSA), and relative apolar surface area (RASA). Alog P is a measure of hydrophilicity or hydrophobicity of a molecule. The logP of non-polar, hydrophobic molecules is highly positive, indicating that they partition into an organic phase. Ghose and Crippen’s approach was used to calculate the AlogP [33].

To investigate the hydrophilic and hydrophobic interactions, the structures of the dispersants and nanoparticles were divided into consistent segments, as they contain groups with varying polarities. Dispersants were represented by an amine repeating unit, that is, the dimethyl amine and hexamer of this group, bis-succinimide, PIB monomer, and PIB_18_ at about 1000 g/mol; functional groups on the insoluble nanoparticle surface were represented by carboxylic acid substituted (Nacid), ketone substituted (Nketone), alcohol (Nalcohol), and dialcohol (Nalcohol2) substituted branched alkane groups as given in DFT calculations. Base oil was represented by the alkyl group with two short branching and one alkene groups (C_24_H_48_), similar to DFT calculations (Figure S1). Calculations indicated that the base oil and PIB groups represented the most hydrophobic entities, exhibiting the highest apolar surface area. The amine group and bis-succinimide groups of dispersants were the most hydrophilic groups that can be coordinated polar groups on the nanoparticle surface (Table 3). It was found that succinimide amine and surface components of the insoluble sludge model are relatively hydrophilic; PIB tails and the base oil matrix were hydrophobic. These findings support the idea that central amine and succinimide groups of the dispersant prefer to interact with the polar surface of the nanoparticle, where the non-polar PIB tail of the dispersant extends into the base oil with similar hydrophobicity.

It should be noted that the sulfonate anionic head group of detergents, which is another oil additive, was also highly hydrophilic with highest relative polar surface area, where the alkyl tail of the same detergents was as hydrophobic as the base oil reported in our previous study [25].

### 3.3. Molecular Dynamics Simulations

Bis-succinimide is more common than tri- or mono- derivatives; thus, polyisobutylene–bis-succinimide was used as dispersant in all calculations. Amine groups were bridged to the PIB tails via succinimide linkage. The straight chain amine group of bis-succinimides is made with polar groups of various diameters and nitrogen contents in industry. Typical polyamine structures at the center are with 6–7 nitrogen and 2.5–3 weight % dispersant in oil. A typical molecular weight of a PIB portion at the two tails is approximately 1000–2000 g/mol. A total of 18 isobutylene repeating units with MW = 1009.90 g/mol and 6 amine groups were used in our model. After the calculation of the free energy of solvation for a single nanoparticle in base oil [25], it was calculated as a value of +32.43 kcal/mol that the nanoparticle did not dissolve in the base oil and aggregate due to polar surface interactions.

**Figure 6:**
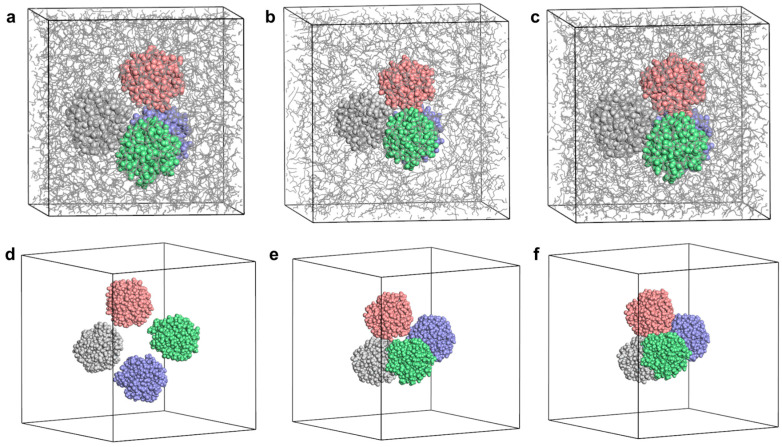
Aggregation of 4 nanoparticles in simulation cell packed with 697 base oil molecules. (**a**) The first, (**b**) middle, and (**c**) final frames of MD simulations. (**d**) The first, (**e**) middle, and (**f**) final frames of MD simulations for aggregated in vacuum for 2 ns simulation time.

Molecular dynamics simulations were initiated by placing nanoparticles into an empty cell followed by in base oil at a size of 8 × 8 × 8 nm^3^, followed by geometry optimization at 5000 steps. The simulation parameters were set as NVT as an ensemble, 423 K as temperature, Nosé–Hoover–Langevin as thermostat with 1 fs step size and 2 ns total simulation time. The first, middle, and the last frames of the molecular dynamic simulation of this cell are given in Figure 6a–c. Nanoparticles have agglomerated not only in base oil, but also in the empty cell.

Initially, the aggregated cluster of four insoluble nanoparticles was placed into an empty simulation cell. Subsequently, the cell containing the aggregated nanoparticles was filled with base oil and subjected to molecular dynamics simulations. In both scenarios, the nanoparticles retained their aggregated structure. The mean square displacement was calculated for both cases. The displacement of the nanoparticles in an empty cell is greater than in base oil (Figure 6). The surfaces of the nanoparticles were polar and hydrophilic, with strong tendency to aggregate both in oil and in vacuum. We concluded that these particles with polar surfaces were highly insoluble in base oil with 24 C that is the main origin of the deposit formation in engines.

**Figure 7:**
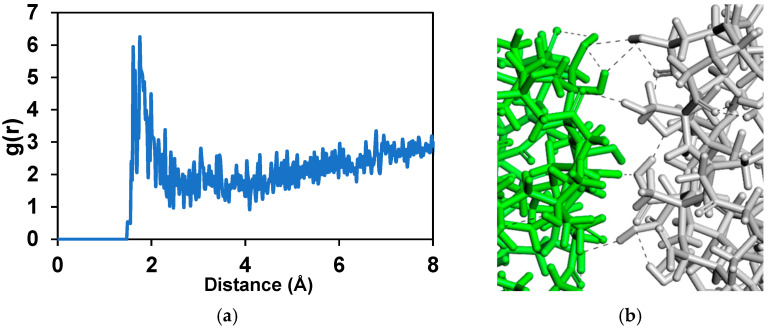
(**a**) Radial distribution function between hydrogen and oxygen atoms of nanoparticles, (**b**) hydrogen bonding between two nanoparticles at equilibrium shown as black dashed lines.

After 5000 geometry optimizations, molecular dynamics simulation was also performed for four nanoparticles positioned in an empty cell center with approximately 4 Å distance. Similarly, quick aggregation behavior was observed. The first, middle, and last frames of the simulation are given in Figure 6d–f for a total of 2 ns simulation. In the absence of any base oil, rapid aggregation was observed, within less than 1 ns of simulation time. Furthermore, it was determined that nanoparticles initially separated by 4 Å gradually approached each other over time, ultimately reaching a distance of 2 Å, as illustrated in the radial distribution function (RDF). The peak at around 2 Å indicates the hydrogen bond formation in theoretical calculations (Figure 7a,b). We concluded that hydrogen bonding between nanoparticles is the second reason for the aggregation mechanism, in addition to the positive free energy of solvation.

**Figure 8:**
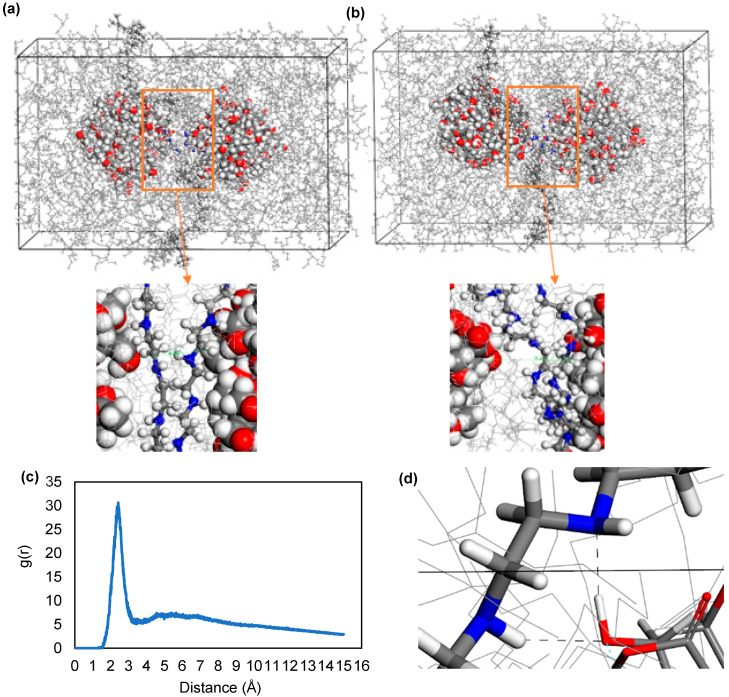
(**a**) The first frame of MD simulations of two nanoparticles with 7 Å distance and two dispersant molecules, (**b**) the last frame of MD simulations of two nanoparticles with 9 Å distance with two dispersant molecules. Inset figures show detailed captures. (**c**) Radial distribution function between amine in dispersant molecules and hydrogen atoms in nanoparticles. (**d**) Hydrogen bonding between nitrogen of dispersant amine group and hydrogen of nanoparticle hydroxyl group shown by black dashed lines.

Further simulations were realized in which two dispersants (Figure 8a,b) were placed in between nanoparticles in different simulations adopted from the lowest energy structures. In these simulations, we observed not only the prevention of the nanoparticles from reaching each other, but also increased distance between them. The observed distance increase between nanoparticles was due to two reasons. First one was the intercalation of the polar amine succinimide group between the nanoparticles, and second was the movement of the longer poly-isobutylene tails of the dispersant molecule in base oil that created shear (Movie S1). We determined that non-polar isobutylene tails were extending into the base oil matrix. RDF analysis clearly showed that hydrogen and oxygen atoms of the nanoparticles have distances of 2 Å with the nitrogen and hydrogen atoms in the amine group of the PIBSI dispersant (Figure 8c,d).

**Figure 9:**
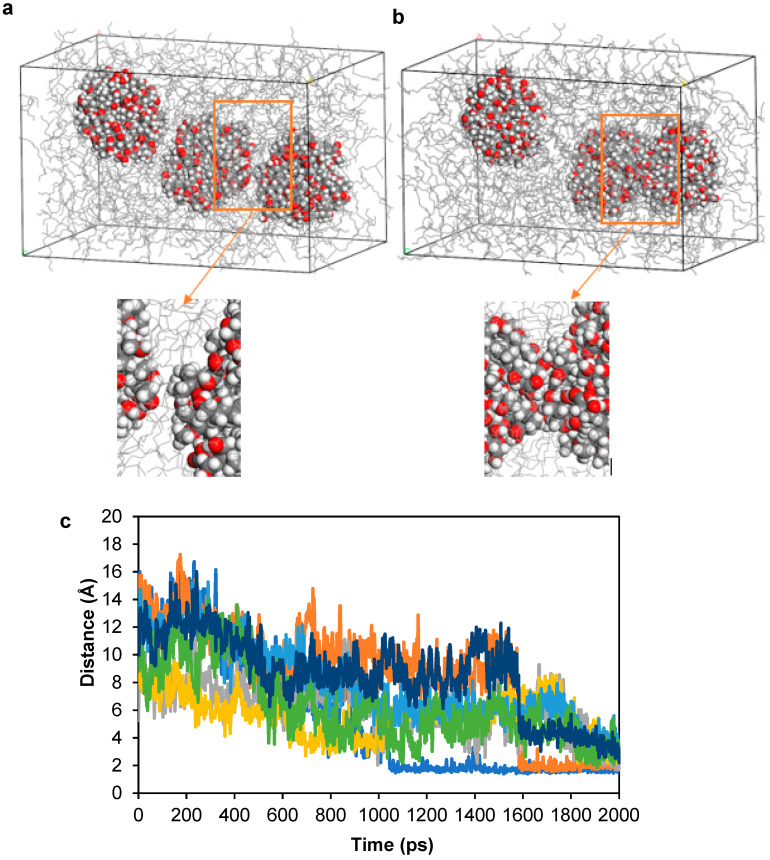
(**a**) The first frame of MD simulations of three nanoparticles with 5 Å distance, (**b**) the last frame of MD simulations of aggregated three nanoparticles. Inset figures show detailed captures. (**c**) Distance evolution of the intermolecular distance between nanoparticles in three-nanoparticle system. Different intermolecular distances between nanoparticles are depicted in different colors.

The new structures were constructed, where the number of nanoparticles increased. Similar with previous simulations, a unit cell with only base oil and nanoparticles was performed first in MD simulations (Figure 9a,b). Randomly selected distances between the surface atoms of the nanoparticles were assessed over a 2 ns simulation period. All evaluated distances exhibited a decreasing trend, with some distances reaching as low as 1.7 Å, as illustrated in Figure 9c. In closer images given in Figure 9, it is shown that hydrogen bonds were formed between aggregated nanoparticles. The distances between aggregated nanoparticles have the highest probability at 1.68 Å distance, which points out strong aggregation behavior.

In order to observe the effect of dispersant addition in the three-nanoparticle system, three, six, and nine dispersant molecules were packed separately into the same cell (Figures S8–S10). For the generated simulation cells in all cases with different numbers of dispersants, the additives were always positioned in such a way that their middle polar part, namely the polar amine bis-succinimide structures, was in interaction with the nanoparticle surfaces. In the MD simulations, this structure was preferred due to the packing calculations that show cells with the lowest energy have the dispersant structures placed in this way. Since the distances between the nanoparticles were relatively small, dispersant molecules were intercalated and interacted with both nanoparticles at the same time (Movies S2–S3).

Mean square displacement analysis for the mobility of the nanoparticles shows that the first system with three dispersant molecules has the highest value (Figure S11). This means that when the number of the dispersant molecules increases, the mobility of the nanoparticles decreases as expected. It was believed that nanoparticles covered by the dispersant form colloid-like structures which do not prefer to aggregate and stay stable in base oil solution.

**Figure 10:**
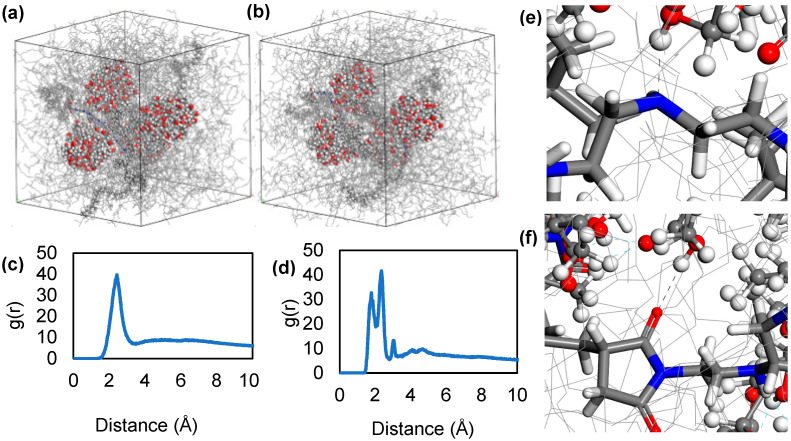
The (**a**) first and (**b**) the last frame of MD simulations of four nanoparticles with four dispersant molecules in 423 K. RDF between hydrogen atoms in nanoparticle surface and (**c**) nitrogen atoms amine portion. (**d**) Oxygen atoms in succinimide portion. Hydrogen bonding between (**e**) the nitrogen atoms in dispersant amine group and hydrogen atoms in nanoparticle surface. (**f**) The oxygen atoms in dispersant succinimide group and hydrogen atoms in nanoparticle surface, shown by black dashed lines.

In order to study the motion of nanoparticles when the dispersant was added, four PIBSI were added to the four-nanoparticle cubic unit cell (Figure 10a,b), and MD simulation was performed again at 423 K temperature which is average engine environment temperature.

For a large number of nanoparticles, even though the distance between these particles was lowered, the dispersants preferred to position themselves between these sludge nanoparticles, which prevented them from forming aggregates. This indicates that the mechanism of the dispersant was not complete separation of the aggregates; rather, it was prevention of the formation of larger aggregates. RDF analysis was performed under the assumption that the amine and succinimide atoms of the dispersant are the ones forming hydrogen bonds with the nanoparticles (Figure 10c,d). Peaks around 2 Å indicated that this estimate was accurate. RDF analysis showed that the amine part is mainly responsible for the interaction of the dispersant with the nanoparticle followed by the succinimide group. Some hydrogen bonds were determined and visualized given in Figure 10e,f.

### 3.4. Coarse-Grained (CG) Simulations

In all-atom MD simulations, we demonstrated that nanoparticle aggregation occurs in the base oil matrix in the absence of dispersant. On the other hand, this aggregation of NPs can be prevented by the interaction of dispersant polar groups with the insoluble nanoparticles followed by the extension of hydrophobic tails into the oil phase that decreases coalesce further by forming a repulsive layer against the other nanoparticles. Moreover, we showed that concentration of the dispersant is an important factor affecting the efficiency of aggregation control. However, all-atom MD simulations are limited to four sludge nanoparticles. The aim of CGMD simulations is to observe the aggregation in a larger simulation cell containing 50 sludge-type nanoparticles. In addition, the effects of other parameters such as the structure and concentration of the dispersant were investigated in CG simulations.

**Figure 11:**
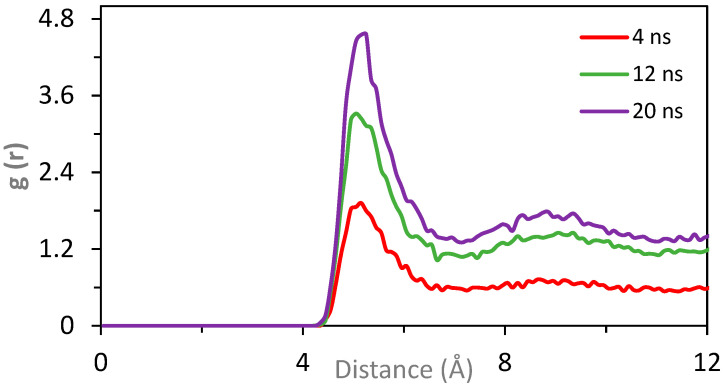
RDF plot for 50 NP (R = 2.2 nm) in 5000 base oil in the absence of dispersant after 4 (red), 12 (green), and 20 ns (purple) of simulation time.

Aggregation of the insoluble deposits occurs due to the attraction between the polar beads present on the surface of the nanoparticles in the absence of dispersant. Polar beads on the surface of the nanoparticles represent polar functional groups present on the surface of the NPs. A 20 ns simulation was conducted, and equilibrium was confirmed based on total energy analysis. Trajectories were recorded every 20 ps, enabling RDF calculations at 4, 12, and 20 ns, which is shown in Figure 11. In the RDF plot shown in Figure 11, the peak value at 5.0 Å is about 1.8 after 4.0 ns simulation time. Equilibrium center-to-center distance of Martini FF-based coarse-grained beads is 4.7 Å for regular beads and 4.3 Å for the ring type of beads (S). Hence, the peak starting from g(r) values of 4.5 and ending at about 6 Å is an indication of a strong interaction between the polar beads on the surface of NPs resulting in aggregation. The RDF peak value continues increasing up to 3.2 and 4.7 Å after 12 and 20 ns of simulation time, respectively. It should be noted that H-bonds could not be observed since these are not atoms but larger beads representing atom groups. While CG models do not explicitly represent hydrogen bonding through directional interactions, such effects can still be captured indirectly via bead parameterization. For instance, polar groups in the Martini force field are assigned interaction parameters that mimic hydrogen bonding tendencies, leading to non-covalent associations between functionalized molecules [34].

**Figure 12:**
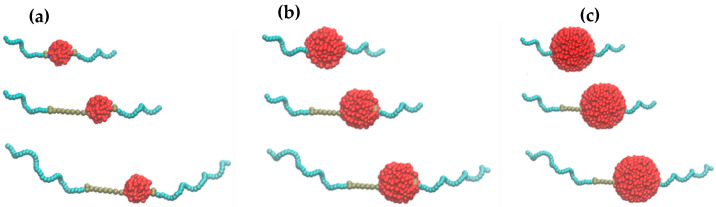
Insoluble deposit models with (**a**) 2.2, (**b**) 3.2, and (**c**) 4.4 nm diameter sizes with dispersant models constitute 6 polar amines at center and two nonpolar PIB tails each with 18 repeat units (top), with 12 polar amines at center and two nonpolar PIB tails each with 18 repeat units (center), and with 12 amines at center and two nonpolar PIB tails each with 36 repeat units (bottom), respectively. Green colored beads represent the PIB tails of the dispersants, brown beads represent the polar beads in amine groups and succinimides, and red color represent nanoparticles.

The dispersant consists of a polar center containing amine groups, which were mapped to the Martini 2.0 force field as P_5_ beads, and two polyisobutylene tails represented as C_1_ beads. Three versions of dispersant were modeled, as shown in Figure 12, to study the effect of size of the amine center and PIB tails on the dispersant. The number of beads in the polar center and the PIB tails is 6 and 18 for Disp v1 (top); 6 and 36 for Disp v2 (center); and 12 and 36 for Disp v3 (bottom).

**Figure 13:**
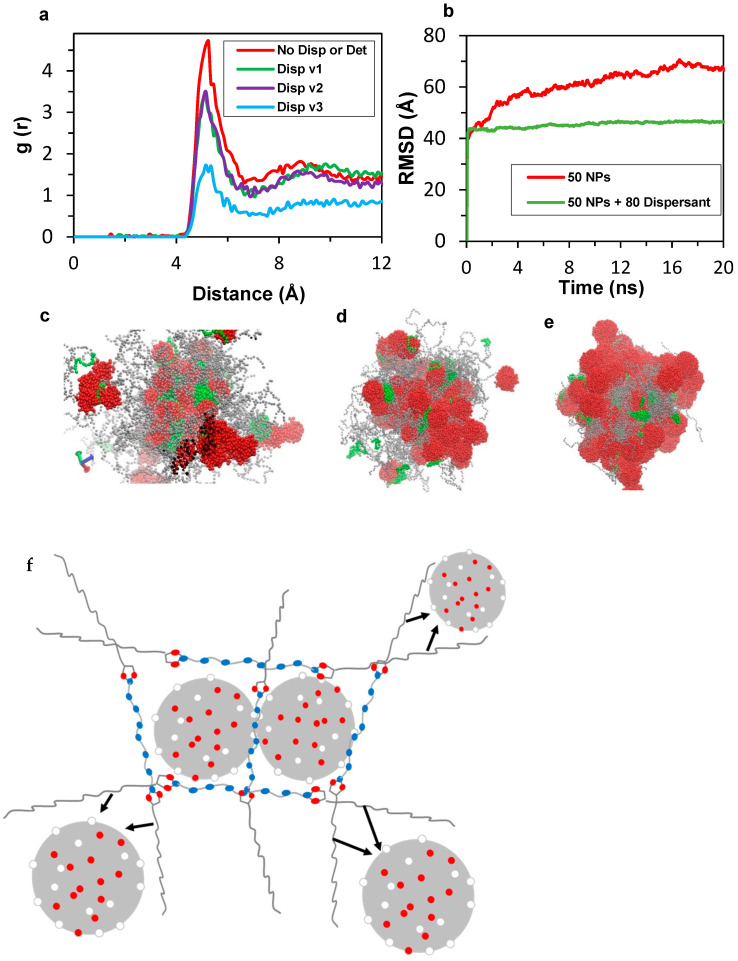
(**a**) RDF plot for 50 NP (R = 2.2 nm) + 5000 base oil, without dispersant (red), with 80 Dispersant v1 (green), with 80 Dispersant v2 (purple), and with 80 Dispersant v3 (blue). (**b**) RMSD plot of 50 NPs having 2.2 nm diameter in 5000 base oil in the absence of dispersant (red) and in the presence of 80 dispersants (green). (**c**) Final snapshot of NPs with 2.2 nm, (**d**) 3.2 nm, and (**e**) 3.2 nm diameter. Polar beads of dispersants (green) intercalate between NPs (red) and apolar tails (gray) create a hydrophobic barrier preventing other NPs approaching in each case. While NP sizes increase, relative size of the dispersant relative to that of NPs decreases, which negatively affects the efficiency of mitigating the aggregation of NPs. (**f**) General mechanism of deposit control by PIBSI dispersant. Arrows represent intermolecular repulsions.

The aggregation of nanoparticles was mitigated by polar amine and succinimide beads in the dispersants. For a simulation cell with small nanoparticles (R = 2.2 nm) in the base oil containing 50 nanoparticles, the RDF peak at ~5 Å decreased from 4.7 (no dispersants) to 3.3 with 80 dispersants composed of 6 polar P_5_ beads and 18 apolar C_1_ beads per molecule. The dispersant’s polar center size had no significant impact, as RDF peaks decreased similarly for both variants with comparable hydrophobic tails, suggesting nanoparticle spacing remained unaffected. Smaller nanoparticle sizes in the simulation may influence this outcome. The extent of the decrease in the RDF peak given in Figure 13a increased when the size of the apolar tails was increased to 36 (as it is 36 in Dispersant v3). The RDF peak value at approximately 5 Å decreases down to a value of 1.7. As can be visually observed in Figure 13c, the non-polar tails of nanoparticles extend in the base oil environment, which creates a hydrophobic barrier and prevents other nanoparticles from aggregating simultaneously. In addition to providing a hydrophobic barrier, extending the non-polar tails inside the base oil matrix restricts the free movement of nanoparticles. This effect is visualized in the RMSD plot given in Figure 13b. It can be observed visually that the noise of the plot is larger in the absence of dispersants, which is an indication of larger fluctuation in the nanoparticle positions. When the diameter of the initial nanoparticles was increased to 3.2 nm and the simulations were repeated, the volumes of the simulation cells were increased and the percentage of nanoparticles by means of the number of beads was increased accordingly. Therefore, it is challenging to compare the results of the simulations by quantitative means. Still, the dispersion mechanism can be explained as given in Figure 13f.

**Figure 14:**
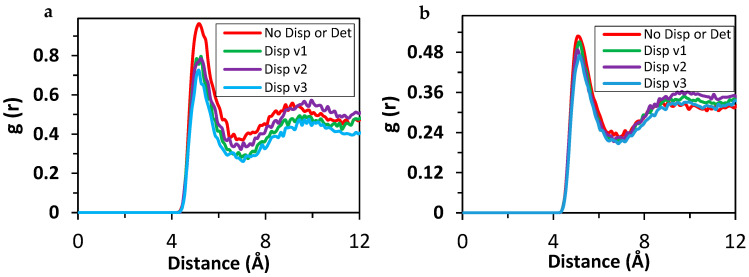
RDF plot for 50 nanoparticles. (**a**) R = 3.2 nm, (**b**) R = 4.4 nm + 5000 base oil, without dispersant (red), with 80 Dispersant v1 (green), with 80 Dispersant v2 (purple), and with Dispersant v3 (blue).

In Figure 14a, the RDF plot for nanoparticles with 3.2 nm is shown. It can be observed from this RDF plot that the extent of aggregation is decreased with use of dispersant for larger nanoparticles. In the RDF plot, the peak value decreased from about 0.96 to 0.78 in the presence of 80 dispersants compared to the case in the absence of dispersants. Similar to the results of smaller-sized nanoparticles, the size of the polar center of the dispersant did not create a remarkable difference by means of peak height. Therefore, the interactions of the surfaces of the nanoparticles were not interrupted more efficiently when the number of polar centers in the dispersants increased. Dispersant v3 with 36 beads in the apolar tail resulted in a slightly higher decrease in the peak intensity at approximately five Å, similar to the case with the nanoparticles of 2.2 nm diameter. In the case of nanoparticles with 4.4 nm diameter, RDF analysis is given in Figure 14b. The difference in the peaks at 5 Å is insignificant compared to the results above. Peak heights decreased in the order of 0.04 by approximately 8%. It is worth noting that the initial configuration is crucial in the final positions in our simulations. In some of the repeated simulations with different initial positions, it was observed that the size of the polar center and/or apolar tail in the dispersant did not have a considerable effect in the aggregation. As discussed above, the volume of the simulation cells increased, and the percentage of nanoparticles by means of the number of beads was increased for larger nanoparticles. This resulted in the decrease in the concentration of the dispersant in the simulation cell, which resulted in an inefficiency in mitigating the aggregation of nanoparticles.

It can be seen in the RDF plots that when the size of the nanoparticles increased, the efficiency of the dispersant for nanoparticle separation decreased. It is clear that the size of the dispersant molecule and hence the hydrophobic barrier created by the apolar tails of the dispersants relative to the size of nanoparticles decreased with that of the nanoparticles, which possibly contributed to the decrease in the efficiency of dispersant.

**Figure 15:**
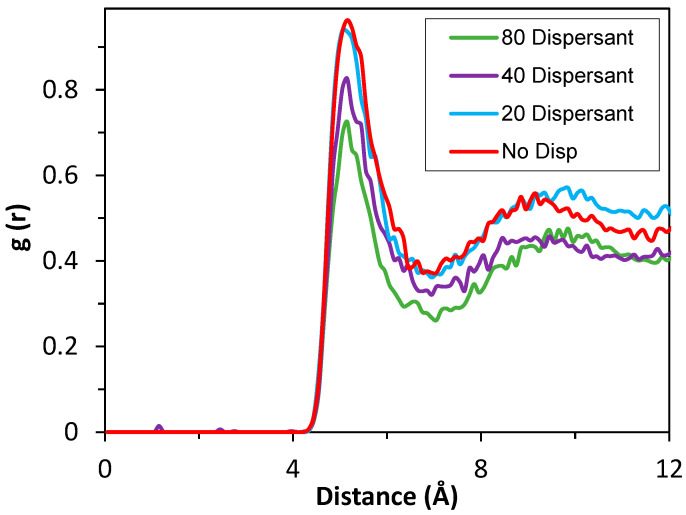
RDF analyses for 50 nanoparticles (r = 3.2 nm) + 5000 base oil, without dispersant (red), with 20 Dispersant (3.22% in the simulation cell), with 40 Dispersant (6.23%), and with 80 Dispersant (11.74%).

Effects of dispersant concentration were studied by RDF analysis, where the addition of 20 dispersants in the simulation cell did not make any difference in the close contact between the 50 aggregated nanoparticles (Figure 15). RDF peak values did not show significant difference compared to the absence of dispersant. The peak of RDF analysis observed at approximately 5 Å decreased gradually from 0.97 to 0.72 in the presence of higher dispersant amount. The number of dispersants in the simulation cell increased up to 40 and 80, where the RDF peak value in the analysis decreased to 0.83 and 0.72, respectively, which demonstrates the importance of additive concentration and its ratio to the sludge concentration on the deposit control mechanism.

## 4. Conclusions

Multiscale modeling methods were used to explain the working mechanisms of a polymer-based PIBSI dispersant, which is one of the main additives used in engine oils to prevent deposit formation. The effect of different groups in dispersants on the deposit control mechanism was successfully examined by multiscale modeling for the first time in the literature. Most probable interactions and configurations of molecules were determined in the interaction energy calculations by DFT methods and solubility parameters based on molecular mechanic methods. The electronic structures of the chemicals in the engine oil, including base oil and dispersant, were determined by first-principle calculations that showed hydrophilic and hydrophobic parts, validating force field-based molecular mechanics calculations.

A sludge model that can aggregate in oil with a relatively polar surface made of carbon and different functional groups was created. Insolubility in the base oil and hydrogen bonds formed between nanoparticles were determined as the origin for nanoparticle aggregation and deposit formation by MD simulations. It was presented that for the cell structures with dispersant molecules that dispersants prevented aggregation formation by entering between nanoparticles. The central amine and succinimide groups of the dispersant intercalate between the nanoparticles and form hydrogen bonds with oxygen atoms which are found on the nanoparticle surface as a result of combustion. Moreover, the hydrophobic PIB tails of the dispersant were extended into the base oil, preventing the aggregation of other nanoparticles. Tail groups extended into the base oil contributed to the dispersion by forming a repulsive layer as well as creating shear by the flow of base oil. It was concluded that the main purpose of the dispersant was not to completely disperse the nanoparticle clusters but to prevent the formation of larger aggregates. Coarse-grained simulations confirmed the MD simulation results at the larger scale, supporting results by all-atom MD simulations. For the increasing size of nanoparticles and fixed number of dispersants, we observed that dispersants were not enough to disperse nanoparticles at high nanoparticle sizes and numbers. Dispersant concentration should be increased for high nanoparticle concentration. Aggregation of nanoparticles is inevitable without any dispersant additives, and both aggregation and aggregate size of nanoparticles can be significantly mitigated by the addition of dispersants.

## Figures and Tables

**Table 1:** Interaction energies for all possible interactions of dispersant units with other constituents.

i	j	ΔE_ij_ (kcal/mol)	i	j	ΔE_ij_ (kcal/mol)
PIB	R-COH	−6.44	PIB	R-(OH)_2_	−7.09
PIB	amine	−8.56	PIB	R=O	−6.54
PIB	bis-suc	−6.62	PIB	R-O-R	−5.65
PIB	R-COOH	−6.82	PIB	PIB	−7.05
PIB	R-OH	−6.99	PIB	sulfonate	−12.88
amine	R-COH	−7.27	amine	R-O-R	−10.25
amine	R-COOH	−8.12	amine	alkyl	−5.76
amine	R-OH	−12.85	amine	amine	−7.79
amine	R-(OH)_2_	−18.88	amine	sulfonate	−22.28
amine	R=O	−7.60			
bis-suc	R-COH	−9.29	bis-suc	R=O	−10.41
bis-suc	bis-suc	−10.63	bis-suc	R-O-R	−8.20
bis-suc	R-COOH	−14.25	bis-suc	amine	−8.87
bis-suc	R-OH	−9.01	bis-suc	sulfonate	−20.68

**Table 2:** Solubility parameters of the base oil, polar (amine and bis-succinimide), and non-polar (PIB) groups on dispersant.

	Van Der Waals Solubility Parameter ((J/cm^3^)^0.5^)	Electrostatic Solubility Parameter ((J/cm^3^)^0.5^)	Total Solubility Parameter ((J/cm^3^)^0.5^)
Base oil [25]	17.013	0.874	17.407
Dispersant (polar center)	20.341	11.233	23.637
Dispersant (non-polar PIB tail)	12.304	0.414	12.671

**Table 3:** Hydrophobicity, SASA, TPSA, TASA, RPSA, RASA for the components of the system.

Structures	AlogP [33]	SASA	TPSA	TASA	RPSA	RASA
Oil molecule [25]	10.47	794.41	0.00	794.41	0.00	1.00
CH_3_COOH	−0.20	212.70	122.97	89.74	0.58	0.42
CH_3_OH	−0.36	170.74	69.06	101.68	0.40	0.60
Nacid	2.71	346.28	76.50	269.77	0.22	0.78
Nketone	2.15	314.80	47.36	267.44	0.15	0.85
Nalcohol	2.14	323.00	29.06	293.94	0.09	0.91
Nalcohol2	1.41	317.06	68.40	248.66	0.22	0.78
Amine center	−1.50	738.57	131.62	606.95	0.18	0.82
Amine monomer	−0.22	209.51	39.09	170.41	0.19	0.81
Bis-succinimide	0.40	322.74	103.50	219.24	0.32	0.68
PIB_18_	25.50	1523.92	0.00	1523.92	0.00	1.00
PIB monomer	2.20	260.08	0.00	260.08	0.00	1.00

## Data Availability

The data presented in this study are available on request from the corresponding author due to the privacy policy of the Lubrizol Corporation.

## Supplementary Materials

The following supporting information can be downloaded at: https://www.mdpi.com/article/10.3390/polym17081041/s1, Movie S1: Sludge particles in the (a) absence and (b) presence of two PIBSI dispersant intercalated between two sludge particles; Movie S2: Amine and bis-succinimide groups at the center of PIBSI dispersant intercalated between sludge particles and polyisobutylene chains are extended into the base oil; Movie S3: Amine and bis-succinimide groups of the PIBSI dispersant intercalated between sludge particles and polyisobutylene chains are extended to form hydrophobic layer; Figure S1: (a) Base oil model used in this study. (b) Dispersant model used in this study; Figure S2: Three simulation cells containing (a) 2 NPs, (b) 2 NPs, and 2 dispersants, (c) 2 NPs and 6 detergents; Figure S3: Three simulation cells containing (a) 3 NPs, (b) 2 NPs and 6 dispersants; Figure S4: Martini model of nanoparticle, consisting of polar, apolar, and non-polar beads; Figure S5: (a) Martini model of base oil, consisting of 7 apolar (C1–C4) beads. (b) Density vs. time plot of the coarse-grained MD simulation of the cell containing only base oil model; Figure S6: Martini model of bis-succinimide dispersant. A total of 36 apolar (C1, cyan) beads for ends, 6 polar (P5, blue) beads in center, 4 polar (P3, P5; gray) and 2 apolar (C3, cyan) beads for rings; Figure S7: Initial cell structures for CGMD study, (a) A total of 50 NPs, (b) 50 NPs and 80 bis-succinimide dispersants; Figure S8: (a) The first frame and (b) the last frame of MD simulations of three nanoparticles with three dispersant molecules; Figure S9: (a) The first frame and (b) the last frame of MD simulations of three nanoparticles with six dispersant molecules; Figure S10: (a) The first frame and (b) the last frame of MD simulations of three nanoparticles with nine dispersant molecules; Figure S11: MSD graphs of three nanoparticles for (a) three, (b) six, and (c) nine dispersant molecule structures; MD simulation movies; CGMD units.

## Abbreviations

The following abbreviations are used in this manuscript:

PIBSI	Polyisobutylene–bis-succinimide
DFT	Density functional theory
MD	Molecular dynamics
CG	Coarse-grained
PIB	Polyisobutylene
PCFF	Polymer consistent force field
LJ	Lennard–Jones
PPPM	Particle–particle mesh solver
PBC	Periodic boundary condition
ESP	Electrostatic potential surface
SASA	Solvent accessible surface area
TPSA	Total polar surface area
TASA	Total apolar surface area
RPSA	Relative polar surface area
RASA	Relative apolar surface area
RDF	Radial pair distribution function
