# Supplementary material for: The Role of Polyisobutylene-Bis-Succinimide (PIBSI) Dispersants in Lubricant Oils on the Deposit Control Mechanism
Source: Polymers (Basel). 2025 Apr 11;17(8):1041. doi: 10.3390/polym17081041 (PMC12030718; doi:10.3390/polym17081041)
Supplement: Supplementary file 1 [file polymers-17-01041-s001.zip › polymers-3562485-supplementary.pdf]

# The Role of Polyisobutylene Bis-Succinimide/PIBSI based Dispersants in Lubricant Oils on the Deposit Control Mechanism

Erhan Özdemir[1], Esra Kan[2], Binbin Guo[3], Eugene Pashkovski[3], Anil Agiral[3]*, Erol Yildirim[1,2,4]*

[1]Department of Chemistry, Middle East Technical University, 06800, Ankara, Turkey.

[2]Department of Polymer Science and Technology, Middle East Technical University, 06800, Ankara, Turkey.

[3]The Lubrizol Corporation, Wickliffe, Ohio, 44092, USA

[4]Department of Micro and Nanotechnology, Middle East Technical University, 06800, Ankara, Turkey.

*Corresponding Author:
anil.agiral@lubrizol.com
erolyil@metu.edu.tr

## SUPPORTING INFORMATION

### *Construction of Base Oil and Dispersant Models*

In the engine oil structure, the base oil has the highest volume ratio. In the structure of the Group II base oil which was used in our model, both small percentage of alkene and branching factors were considered. The average carbon number of isoparaffins and naphthenic groups is between 20 and 25. According to this information, base oil was modeled as a branched hydrocarbon structure having 24 C atoms. The experimental density for the base oil is 0.86-0.87 g/cm$^3$. An alkene group at the center with short branching is preferred in order to reach the desired density. The structure of the base oil model used in this study is shown in Figure S1a. The base oil is the major constituent of the lubricant additive according to the experimental results provided by the company. The same density with the experimental value was calculated in MD simulations in constant pressure with the cell containing only base oil. Dispersant having PIB tails containing 18 isobutylene units and polar amine groups in the center, connected with bis-succinimide was modeled as shown in Figure S1b.

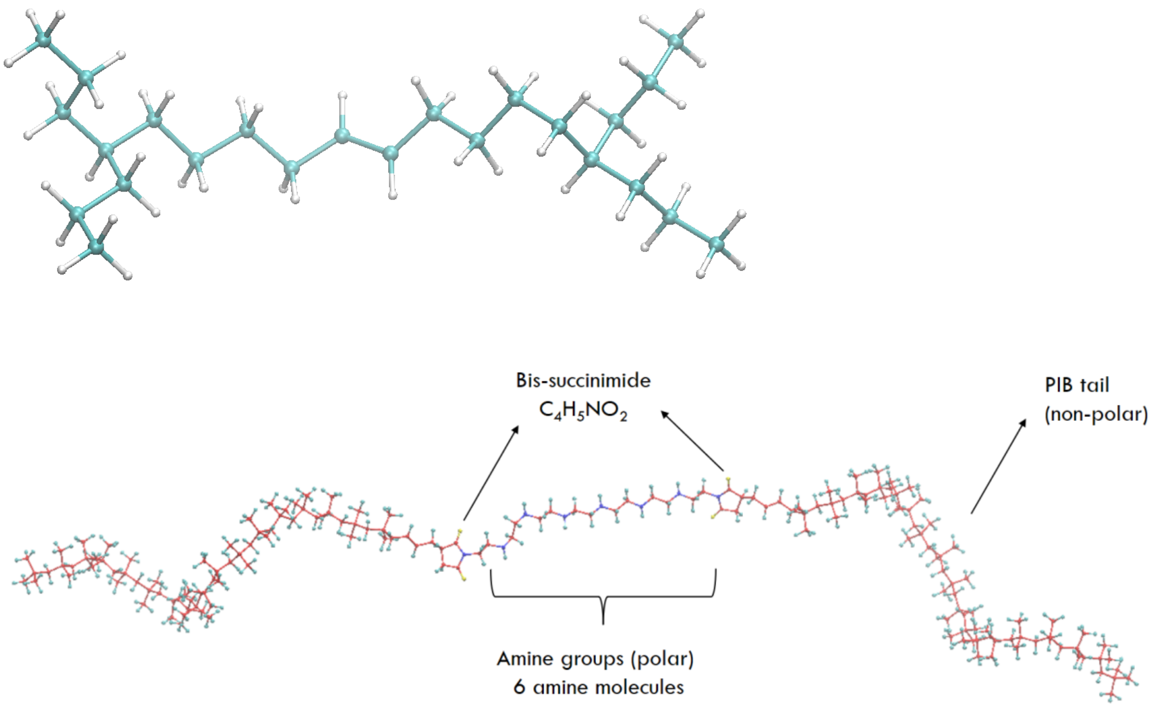


a

b

**Figure S1.** a) Base oil model used in this study. b) Dispersant model used in this study.

***Construction of the Simulation Cell***

For all-atom molecular dynamics simulations, simulation cells were prepared with Scienomics MAPS 4.4, simulations were performed in LAMMPS (3 Mar 2020 version) and visualized by MAPS 4.4 and VMD. Scienomics MAPS 4.4 was also used to apply the PCFF force field to the atoms in the constructed periodic models.

In the first part, three simulation cells containing two insoluble nanoparticles were prepared as shown in Figure S2. No dispersant was added to the first cell. In the second cell, two dispersants were added in the center of the simulation cell, in between two insoluble NP models. The positions of the dispersants were chosen using Monte Carlo simulations. All the cells were filled with base oil.

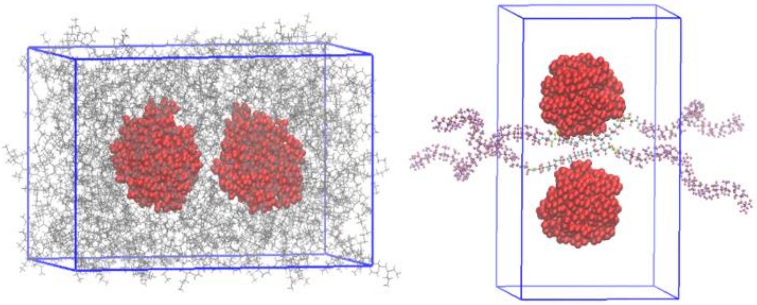

**Figure S2**. Three simulation cells containing a) 2 NPs, b) 2 NPs and 2 dispersants. Base oil molecules are hidden in the second and third cell for simplicity.

Simulation cells containing three nanoparticles were also prepared similarly as shown in Figure S3. No dispersant or detergent was added to the first cell. In the second cell, three and six dispersants were added in the center of the simulation cell, respectively. All the cells were filled with base oil.

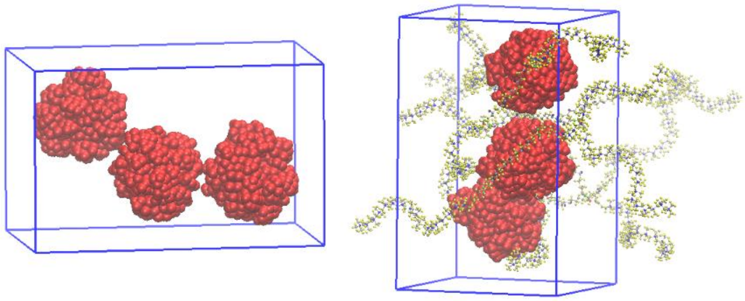

**Figure S3.** Three simulation cells containing a) 3 NPs, b) 3 NPs and 6 dispersants. Base oil molecules are hidden for simplicity.

***Construction of the Insoluble Deposit Model for CGMD simulations.***

The Scienomics Atomeso Converter was employed to map the atoms of the deposit model onto coarse-grained (CG) beads for CGMD simulations using the Martini 2.0 force field. The insoluble nanoparticle, with a diameter of 2.2 nm, comprised 123 CG beads. To reconstruct the nanoparticle model, Packmol software was used to randomly place the beads within the specified spatial intervals similar to the mapped insoluble deposit model. This process ensured that the distribution

reflected the correct bead types and their densities, maintaining the intended composition and structural characteristics of the nanoparticle. A Python script was utilized to assign bonds randomly between the beads, beginning from the center bead and ensuring that each bead forms at least one and at most six bonds to conserve their integrity and shape. The beads should keep their spherical structure during the simulations to meet the experimental results. Shortly, spherical structure is a convenient starting point to model large and porous deposit formation. By using the same method, nanoparticles having diameters of 3.2 and 4.4 nm were prepared using Packmol software and Python script. The number of beads were increased proportional to the extension in the volume. Nanoparticles having 3.2 nm diameter contain 383 beads and those having 4.4 nm diameter contain 984 beads. Deposit models constructed for coarse-grained MD simulations are shown in Figure S4.

Moltemplate package was utilized to prepare cells for simulations in LAMMPS package. To verify that the nanoparticles remained in their spherical form throughout the simulation, 10 ps long NPT followed by 10 ps NVT simulations were performed until a NP with a satisfactory shape was obtained.

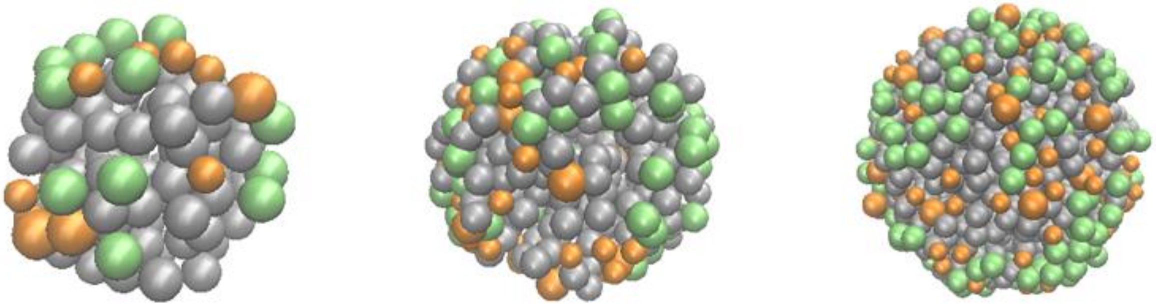

**Figure S4.** Martini model of nanoparticle, consisting of polar, apolar and non-polar beads. Total number of beads are 123, 383 and 984, respectively. Images are resized to fit in-line.

### *Construction of the Base Oil Model*

Base oil was mapped consisting of seven apolar beads at different levels of polarity. The central bead containing the only alkene group in the structure was mapped to C4 apolar bead. Branching alkanes on the left and right sides of the structure were mapped as C3 and the beads connecting

central bead to the branch beads were represented as C1 beads as shown in Figure S5a. CGMD simulations in NPT ensemble were also run to check the density of coarse-grained base oil model. As shown in S5b, a satisfactory value of density, which is in between 0.86 and 0.87 g /cm$^3$, was obtained quickly. This important step validated our model and simulation methodology.

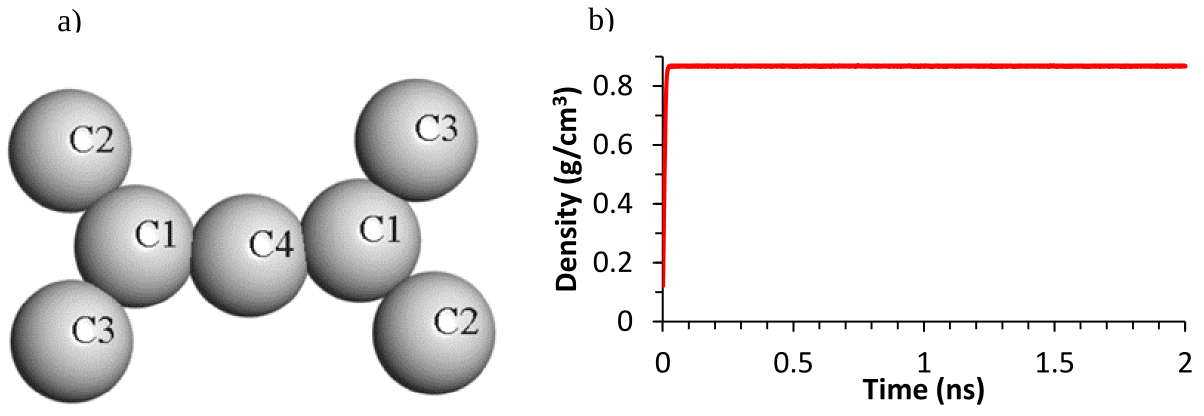


**Figure S5.** a) Martini model of base oil, consisting of 7 apolar (C1-C4) beads. b) Density vs. time plot of the coarse-grained MD simulation of the cell containing only base oil model.

***Construction of the Dispersant Model***

Polyisobutylene-bis-succinimide (PIBSI) type dispersant was mapped into Martini coarse-grained force field consisting of three chemically different components as it is in the all-atom model. Polar $P_5$ beads depict the central amine group while $P_3$-$P_5$ couples represent the succinimide groups on either side of the amine group. These beads are connecting the amine group to two apolar tails as shown in Figure S6.

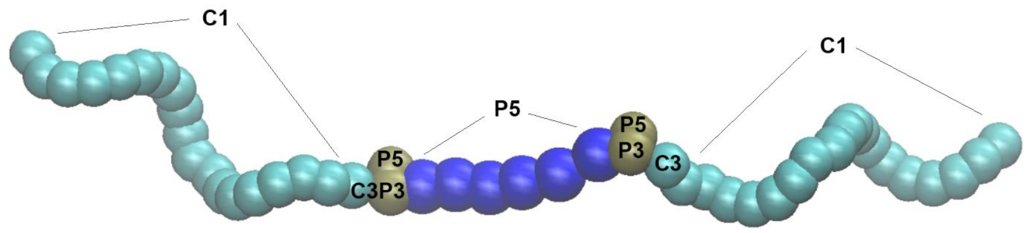


**Figure S6.** Martini model of bis-succinimide dispersant. 36 apolar ($C_1$, cyan) beads for ends, 6 polar ($P_5$, blue) beads in center, 4 polar ($P_3$, $P_5$; gray) and 2 apolar ($C_3$, cyan) beads for rings.

### *Construction of the Simulation Cell*

To reproduce and analyze molecular details of the experimental results by computational methods, a simulation cell was prepared with 50 NPs, 5000 base oil and 80 dispersant having 12 polar center beads and 36 tail beads. The number of the dispersant molecules were decreased to 40, 20 and 0 by simply deleting existing dispersants randomly from the first simulation cell so initial positions of the NPs, base oils and dispersants existing in the simulation cell were the same in these four simulation cells. Within the simulation cell with 80 dispersants, the dispersants constitute 11.74% of the cell based on the number of beads present. Simulation cells having 40 and 20 dispersant constitute 6.23% and 3.22% of the cells, respectively.

For CG MD simulations, ten of the insoluble deposit models were inserted in the center of the simulation box and the remaining 40 NPs were inserted into the surrounding as shown in Figure S7. Throughout the CGMD simulations, aggregation of the nanoparticles was monitored from RDF analysis between the polar groups of the central 10 NPs and those of the surrounding 40 NPs. It is important to consider that, the positions of the NPs at the end of the simulation depends strongly on the initial configurations. Hence, the trajectory files were also visualized by eye to check whether the aggregation between the central beads and the surrounding beads was a good representation of the aggregation inside the whole cell.

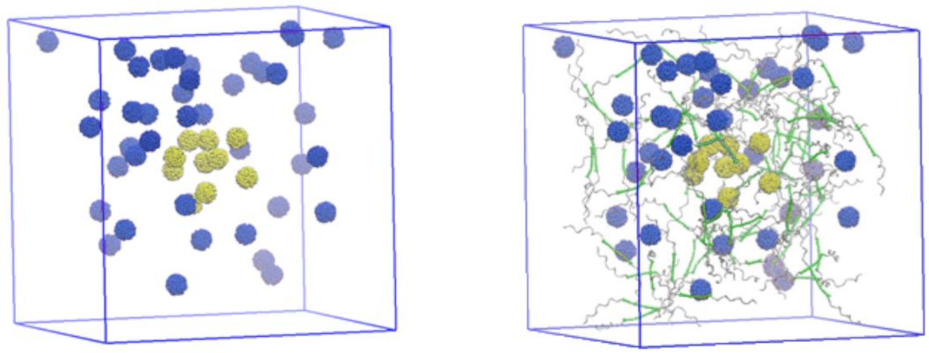

**Figure S7.** Initial cell structures for CGMD study, a) 50 NPs, b) 50 NPs and 80 bis-succinimide dispersants. 5000 base oil molecules are hidden for simplicity.

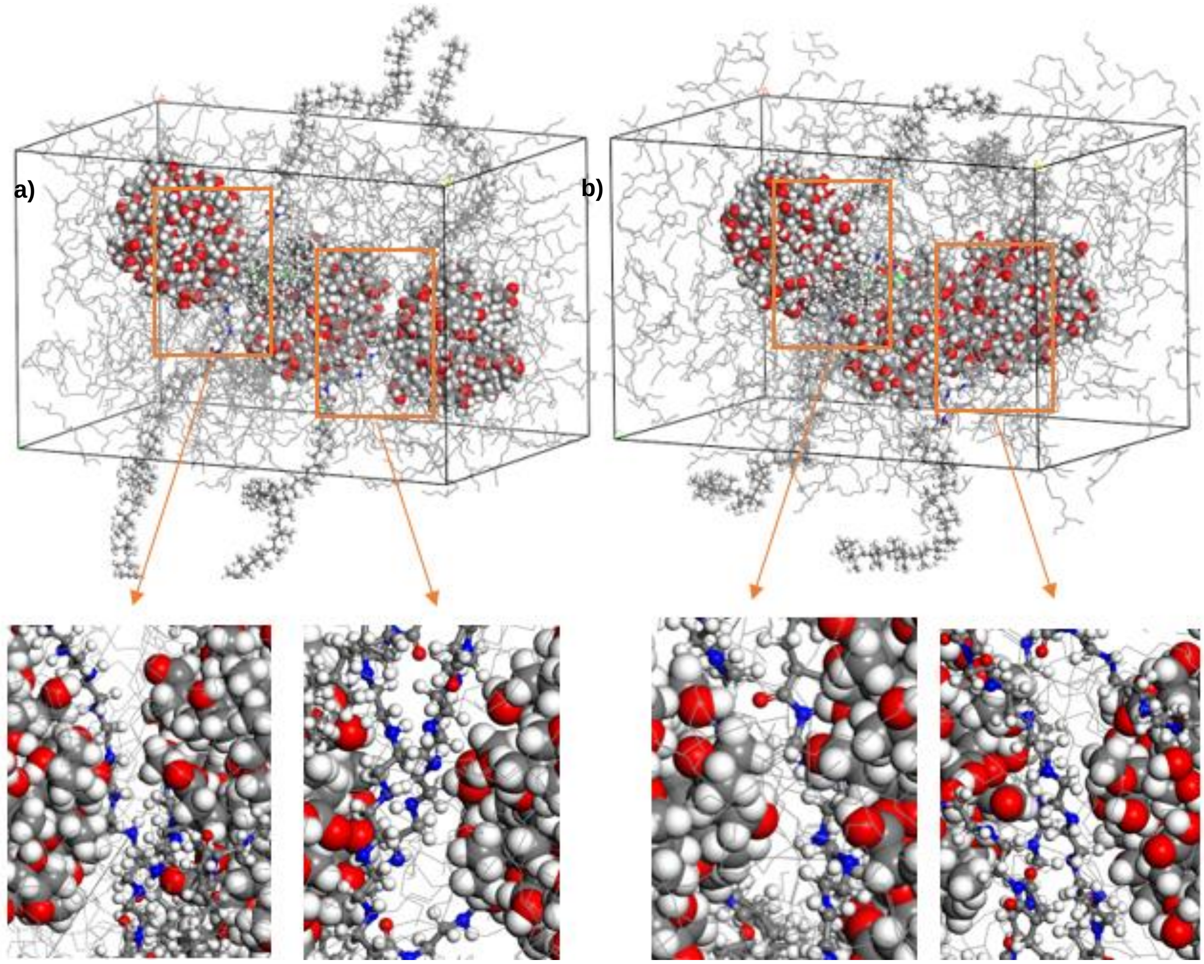


**Figure S8.** a) The first frame and b) the last frame of MD simulations of three nanoparticles with three dispersant molecules. Inset figures show detailed captures.

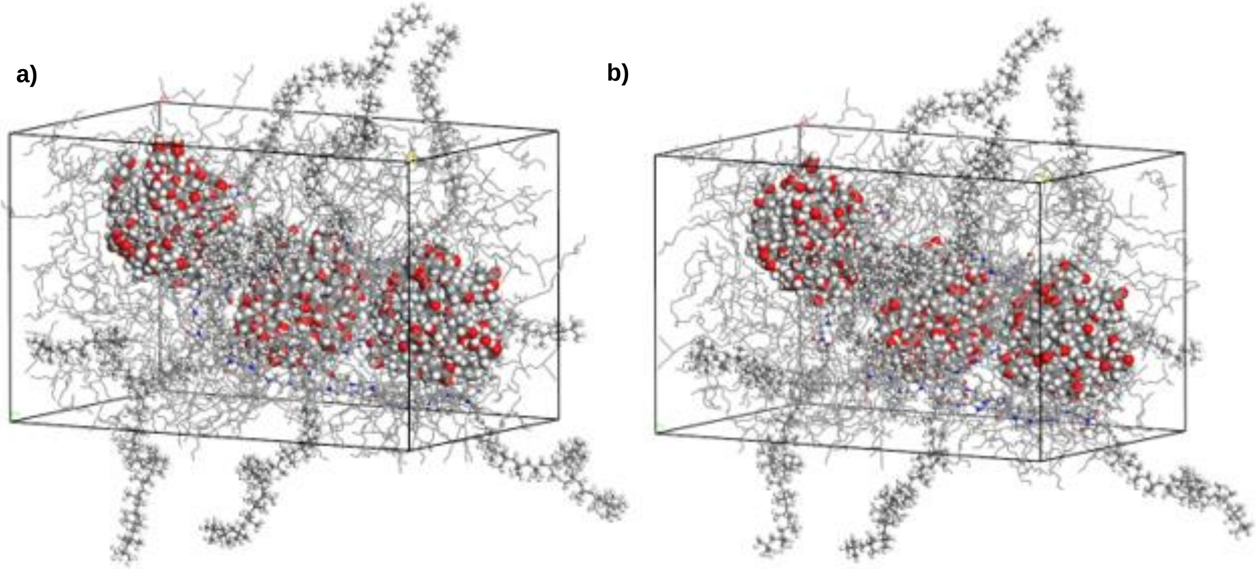

**Figure S9**. a) The first frame and b) the last frame of MD simulations of three nanoparticles with six dispersant molecules.

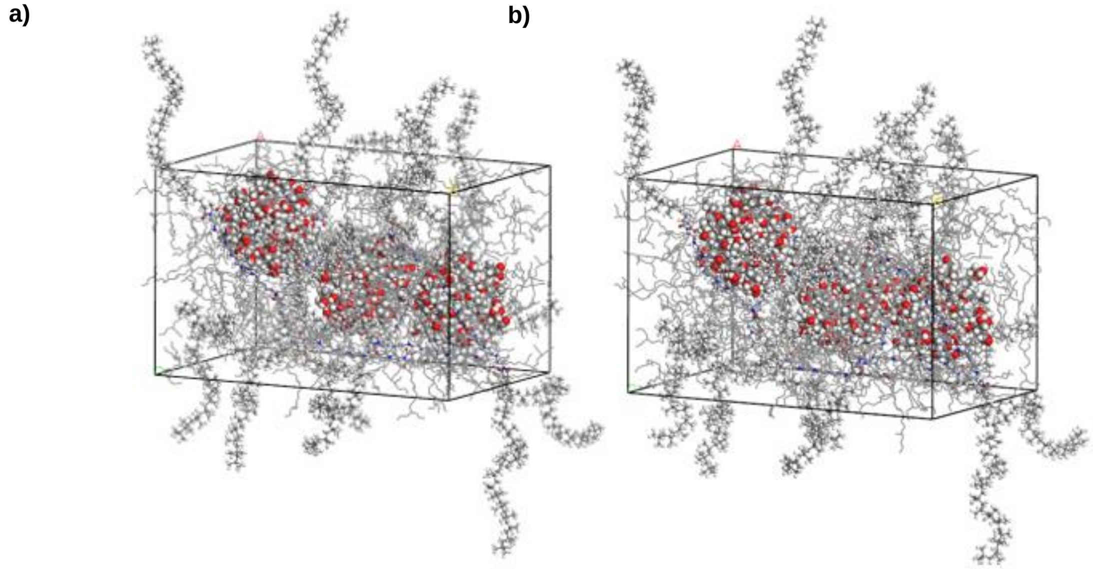


**Figure S10**. a) The first frame and b) the last frame of MD simulations of three nanoparticles with nine dispersant molecules.

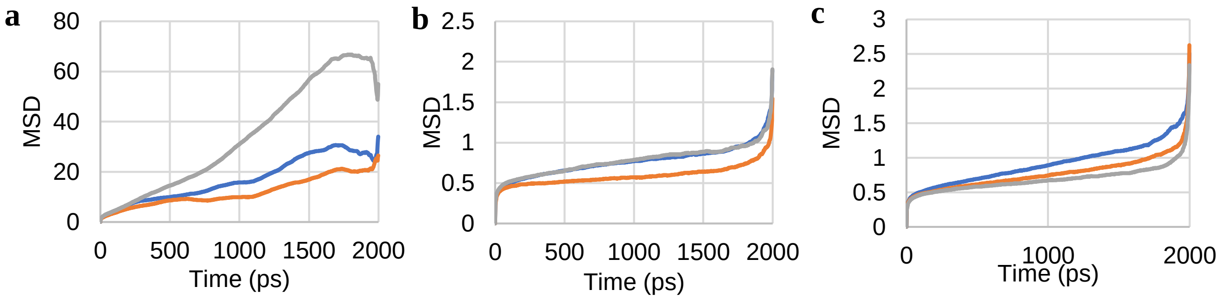


**Figure S11**. MSD graphs of three nanoparticles for a) three, b) six, and c) nine dispersant molecule structures.
