# Supplementary material for: The Role of Polyisobutylene-Bis-Succinimide (PIBSI) Dispersants in Lubricant Oils on the Deposit Control Mechanism
Source: Polymers (Basel). 2025 Apr 11;17(8):1041. doi: 10.3390/polym17081041 (PMC12030718; doi:10.3390/polym17081041)
Supplement: Supplementary file 1 [file polymers-17-01041-s001.zip › MovieS2.pptx]

## Slide 1
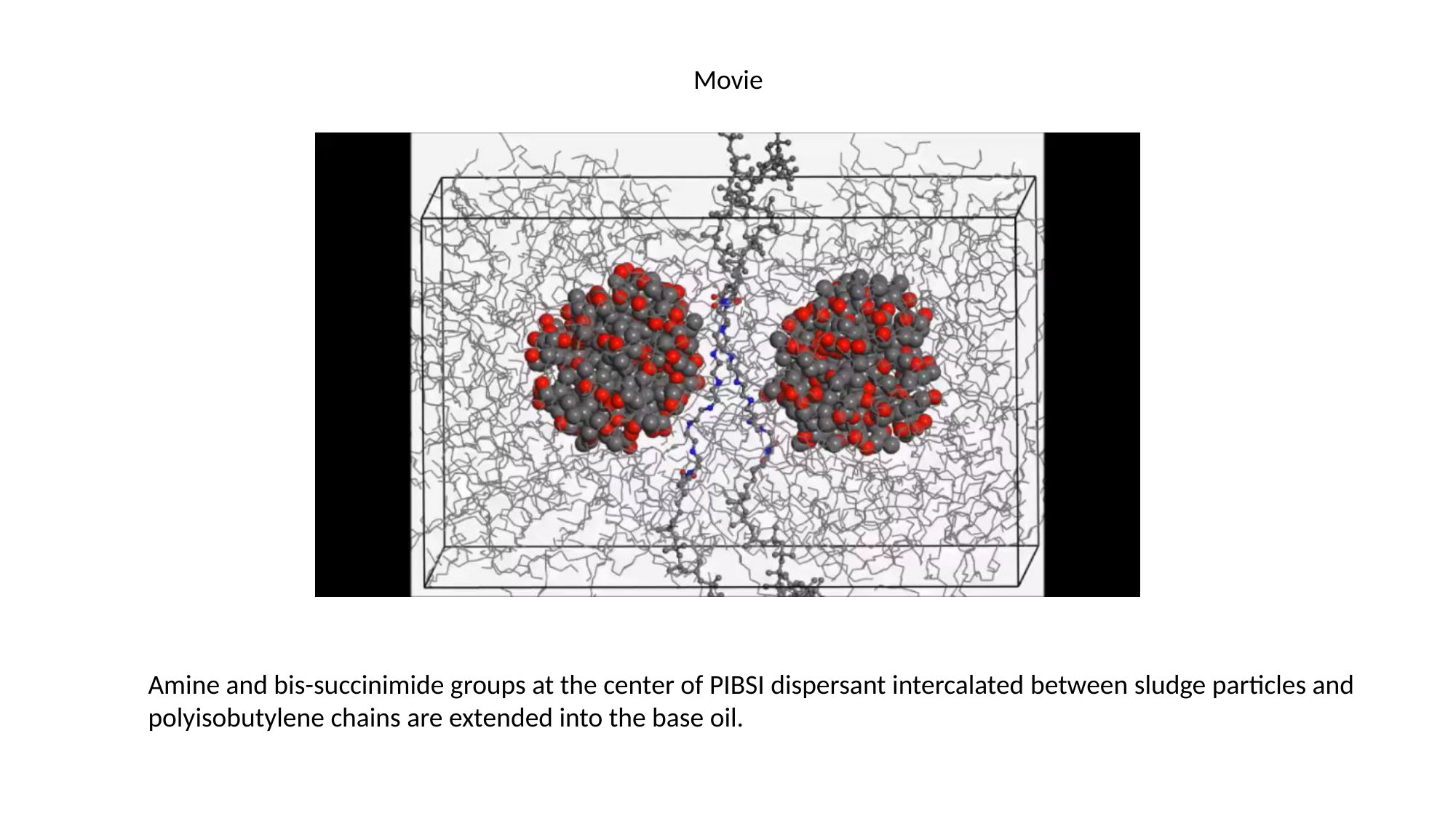

Movie
Amine and bis-succinimide groups at the center of PIBSI dispersant intercalated between sludge particles and polyisobutylene chains are extended into the base oil.
